# Plasma β-III tubulin, neurofilament light chain and glial fibrillary acidic protein are associated with neurodegeneration and progression in schizophrenia

**DOI:** 10.1038/s41598-020-71060-4

**Published:** 2020-08-31

**Authors:** Daniela Rodrigues-Amorim, Tania Rivera-Baltanás, María del Carmen Vallejo-Curto, Cynthia Rodriguez-Jamardo, Elena de las Heras, Carolina Barreiro-Villar, María Blanco-Formoso, Patricia Fernández-Palleiro, María Álvarez-Ariza, Marta López, Alejandro García-Caballero, José Manuel Olivares, Carlos Spuch

**Affiliations:** 1 Translational Neuroscience Research Group, Galicia Sur Health Research Institute, University of Vigo, CIBERSAM, Vigo, Spain; 2 Departement of Psychiatry, University of Santiago de Compostela, Santiago de Compostela, Spain; 3 Hospital Álvaro Cunqueiro, Bloque Técnico, Galicia Sur Health Research Institute – IISGS, Planta 2, Sala de Investigación, Estrada Clara Campoamor, 341, 36212 Vigo, Spain

**Keywords:** Schizophrenia, Predictive markers

## Abstract

Schizophrenia is a progressive disorder characterized by multiple psychotic relapses. After every relapse, patients may not fully recover, and this may lead to a progressive loss of functionality. Pharmacological treatment represents a key factor to minimize the biological, psychological and psychosocial impact of the disorder. The number of relapses and the duration of psychotic episodes induce a potential neuronal damage and subsequently, neurodegenerative processes. Thus, a comparative study was performed, including forty healthy controls and forty-two SZ patients divided into first-episode psychosis (FEP) and chronic SZ (CSZ) subgroups, where the CSZ sub group was subdivided by antipsychotic treatment. In order to measure the potential neuronal damage, plasma levels of β-III tubulin, neurofilament light chain (Nf-L), and glial fibrillary acidic protein (GFAP) were performed. The results revealed that the levels of these proteins were increased in the SZ group compared to the control group (*P* < 0.05). Moreover, multiple comparison analysis showed highly significant levels of β-III tubulin (*P* = 0.0002), Nf-L (*P* = 0.0403) and GFAP (*P* < 0.015) in the subgroup of CSZ clozapine-treated. In conclusion, β-III tubulin, Nf-L and GFAP proteins may be potential biomarkers of neurodegeneration and progression in SZ.

## Introduction

**Figure 1:**
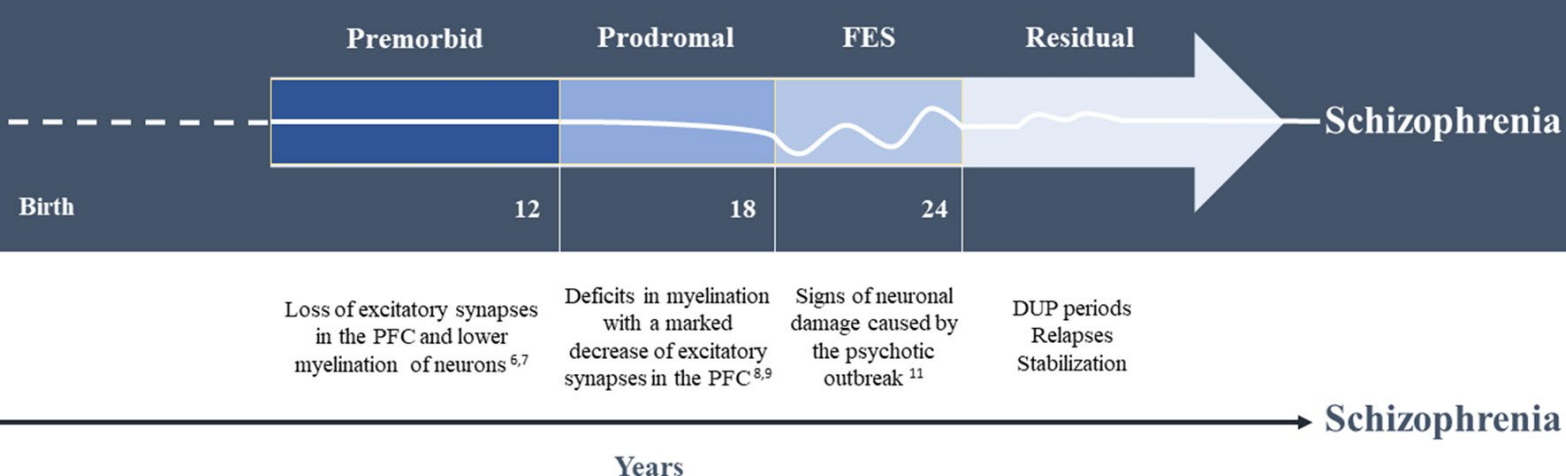
Clinical course of schizophrenia. PFC: prefrontal cortex; FEP: first-episode of psychosis; DUP: duration of untreated psychosis.

Schizophrenia (SZ) is a complex, severe and heterogeneous disorder. Unfortunately, its aetiology and pathophysiology remains unclear^1^. Although schizophrenia can occur at any age, the average age of onset tends to be in the late teens to the early 20s for men, and the late 20s to early 30s for women, and its deteriorating course makes SZ the most disabling psychiatric disorder^2^. SZ has been explained both by neurodevelopmental and neurodegenerative models, which include neuronal damage and grey matter abnormalities^3,4^. Both models may be complementary, and four stages can be proposed in the course of SZ^5–11^ (Fig. 1). Moreover, the duration of untreated psychosis (DUP) is a critical period during schizophrenia course. Accumulating evidence suggests that a longer DUP is associated with clinical deterioration, including increased symptoms severity, cognitive and functioning declines, and poorer response to antipsychotics^12,13^. SZ patients treated immediately after the FEP will suffer less cognitive impairment and disability^14–17^.

Cognitive deficits are considered part of the core symptoms of SZ^18^. Both abnormalities in prenatal life and in the early childhood neurodevelopment are associated with SZ, and they may explain the early cognitive decline that characterizes this disorder^8,19^. Pathological neurodevelopment described in SZ patients may be explained by the disruption of neurogenesis and specific molecular signal abnormalities^4^. Both genetic and environmental factors play a crucial role in this early pathological neurodevelopment^20,21^. Therefore, neurodevelopment hypothesis involves genetics and exposure to environmental risk factors such as obstetric complications, season of birth, maternal malnutrition, pre and postnatal infection, urbanization, migration, risk behaviours, among others^22–24^. Taking into account that the onset of SZ starts long before the appearance of the FEP, it would be expected that during the prodromal stage of SZ some biomarkers would appear, indicating neurodegeneration. This has been already described in neuroimaging studies, showing structural changes in the brain in high-risk for psychosis persons^25^. In these studies, both in high-risk individuals and in early-onset patients, smaller brain volumes can be seen, especially in frontotemporal areas^26,27^. This reduction in gray matter is progressive and more pronounced in certain areas, such as the anterior cingulate, frontal lobe (medial and inferior), temporal lobe, hippocampus, amygdala, thalamus and the insula^28^.

Antipsychotics are the main treatment for SZ. However, although the effectiveness of antipsychotic treatments has been clearly established, the effect of their chronic use in SZ patients has been questioned^29^. There are important differences in side-effects profiles between first- and second-generation antipsychotics (FGAs and SGAs), while both classes have comparable efficacy on positive symptoms^30^. For example, clozapine may induce agranulocytosis, so it is reserved for those patients who do not respond to other antipsychotics^31^. Recently, *Ibi *et al. have described the mechanisms by which the chronic use of antipsychotics may cause cognitive damage. In this study, they claim that the blocking of the 5HT2A serotonin receptor induced by some antipsychotic drugs activates inflammatory pathways in the brain^32^. However, literature review shows that haloperidol exerts neurotoxic effects at any dose, leading to neuronal death through different molecular mechanisms^33^, while SGAs present neuroprotective effects (dose-dependent) due to their multiple molecular mechanisms^34^. If this were the case, the neuroprotective effects of SGAs could prevent and restore the neurodegenerative effects of psychosis^34^.

On the other hand, for over 100 years, even before antipsychotic treatments were available, SZ has been discussed as a degenerative disorder^3^. The neurobiology of SZ is based on disruptive processes in neurons, neurotransmitters, synapsis, and axonal integrity, whose functions are essential to brain networks connectivity^20,35,36^. Moreover, structural proteins that are part of the neurons’ cytoskeleton contribute to the neuroplasticity by dynamic processes such as the morphology of dendrites, synapsis formation, neuronal migration and differentiation^37,38^. Consequently, abnormalities in the cytoskeleton, as well as neuronal death (axonal disintegration), were correlated with the pathophysiology of SZ^37,39^. In this sense, proteins such as β-III tubulin (microtubule of dendrites and axons), neurofilament light chain (Nf-L) (structural protein exclusive of neurons), and glial fibrillary acidic protein (GFAP) (structural component of astrocytal cytoskeleton) which are associated with neurodegenerative diseases such as Alzheimer or amyotrophic lateral sclerosis (ALS)^38–41^ may have a substantial role in SZ. Furthermore, these proteins can be detected in peripheral samples^39^. In the last 5 years, proteomics has been used in the search for potential biomarkers in peripheral blood, and abnormal levels of tubulin in patients with SZ have been described elsewhere^42,43^.

In the present study, we will focus on three structural proteins (β-III tubulin, Nf-L and GFAP) that are associated with neurological impairment and clinical progression of neurodegenerative disorders, with the aim of establishing a pattern of neurodegeneration in patients with SZ.

## Methods

### Subjects and plasma sampling

Blood samples were obtained in vacuum tubes with K_2_EDTA from 42 SZ patients hospitalized at the Psychiatry Department of Álvaro Cunqueiro Hospital in Vigo, Spain, between 7:00 and 9:00 h. The diagnosis of SZ was established by psychiatrists according to the Diagnostic and Statistical Manual of Mental Disorders (DSM-5). Nine patients were diagnosed of FEP (drug näive), and 33 patients were CSZ treated with aripiprazole (*n* = 8), risperidone (*n* = 8), olanzapine (*n* = 8) or clozapine (*n* = 9). Subgroups of CSZ were made, based on the antipsychotic’s treatment (in monotherapy). Each SZ patient had been treated with only one antipsychotic (risperidone, aripiprazole or olanzapine), except clozapine-treated patients, who had not responded before to two different types of antipsychotics (Supplementary Table 1). The limited number of participants per group was due to the difficulty of including patients on antipsychotic monotherapy. Inclusion criteria were: patients aged 18 years or older, who provided the signed informed consent, agreeing with Helsinki Declaration and the approval of the Ethics Committee (*Galician Network of Research Ethics Committees*). Individuals with other psychiatric or neurological disorders, traumatic brain injury, diabetes or history of substance abuse were excluded from the study. Pregnant or breastfeeding women were also excluded. Concomitant medication of SZ patients included angiotensin converting enzyme inhibitors (ACE inhibitors), stains, analgesics (paracetamol), benzodiazepines (lorazepam) or levothyroxine (hypothyroidism). Blood samples from 40 healthy controls were also collected in the Álvaro Cunqueiro Hospital under the same conditions, providing also the informed consent signed. Information was collected about age, gender, illness onset, duration of illness, and antipsychotic treatment. Levels of lymphocytes and neutrophils were recollected from clinical records. Patient groups were matched by age and gender with healthy controls. The positive and negative syndrome scale (PANSS) was applied to evaluate the level and intensity of positive and negative symptoms, and general psychopathology of patients with schizophrenia. Clinical and demographic characteristics of participants are showed in Table 1.

**Table 1:** Demographics.

Parameter	Schizophrenia					Controls	*P* value
Total number (N)	42					40	–
Age (years)	41.10 ± 14.41					44.96 ± 14.95	0.3030^a^
Gender (M/F)	26/16					25/15	1.0000^b^
Illness onset (years)	22.36 ± 15.34					–	0.0556^c^
Duration of illness (years)	11.95 ± 10.58					–	0.8974^c^
Antipsychotic drugs	FEP	Risperidone	Olanzapine	Aripiprazole	Clozapine	–	–
Number (N)	9	8	8	8	9	–	–
Dose mg	–	4.00 ± 0.68	12.50 ± 1.34	22.38 ± 4.19	322.22 ± 109.29	–	–
Dose equivalents mg-clozapine	–	400,00 ± 68,13	212,50 ± 27,95	298,33 ± 55,83	322.22 ± 109.29	–	0.0870^c^
PANSS							
PANSS Positive	24,00 ± 1.68	18.00 ± 3.42	17.25 ± 2.22	17.38 ± 1.77	21.44 ± 2.03	–	0.1543^d^
PANSS Negative	27.22 ± 2.85	27.00 ± 4.08	26.25 ± 2.46	32.88 ± 2.09	35.11 ± 1.44	–	0.0799^d^
PANSS General	41.22 ± 3.29	29.13 ± 2.52	30.25 ± 1.86	29.25 ± 1.83	39.44 ± 1.26	–	0.0003^d^

Mean ± SD. ^a^Mann-Whitney U test *P* value; ^b^Fisher’s exact test *P* value. ^c^One-way ANOVA test *P* value followed by Bonferroni post-hoc test among groups of patients treated with antipsychotics.^d^One-way ANOVA test *P* value followed by Bonferroni post-hoc test among schizophrenic groups.

FEP: first-episode psychosis. PANSS: positive and negative syndrome scale, PANSS positive and negative ranges: 7–49; PANSS general range: 16–112; PANSS total range: 28–210.

*Statistical significance: *P* ≤ 0.05.

### Plasma sample preparation

Venous blood was collected in two vacuum tubes with K_2_EDTA (BD vacutainer; Becton, Dickinson & Co., USA) and was centrifuged at 1000×*g* for 10 min. Plasma was separated and stored in aliquots at − 80 °C. To perform the analysis, plasma total proteins were measured by a bicinchoninic acid assay (BCA, Pierce Chemicals, Rockford, IL).

### Quantitative immunoblotting

Aliquots of plasma of patients and controls were prepared by addition of Laemmli buffer 2x (Bio-Rad, California, USA), and boiled at 95 °C for 5 min. Plasma proteins previously measured (total proteins) allowed to determine the equivalent amount of proteins required for each sample (10 µg of proteins) (Supplementary Table 2). Fraction volumes were loaded in 6–8% Bis–Tris polyacrylamide gels and electrophoresis was performed in PowerPac™ universal power supply (Bio-Rad, California, USA) at 60 V for 30 min, and 120 V for 90 min. Proteins were immediately transferred to polyvinylidene difluoride (Immun-Blot^®^ PVDF membrane, Bio-Rad, California, USA), using PowerPac™ universal power supply (Bio-Rad, California, USA) at 0.25 A for 60 min (for two gels). Membranes were blocked with 5% milk in tris buffer saline solution with Tween (TBST) for 20 min and washed three-times with TBST. The membranes were then incubated overnight at 4 °C over stirrer with primary antibody: anti-tubulin, β-III isoform, mouse monoclonal antibody 1:1,000 (MAB1637, Millipore, Massachusetts, USA); anti-Nf-L mouse monoclonal antibody 1:1,000 (900-301-D83, Rockland Immunochemicals Inc., Limerick, Ireland); or anti-GFAP mouse monoclonal antibody 1:1,000 (200-301-W55, Rockland Immunochemicals Inc., Limerick, Ireland). After washing them three-times with TBST, the membranes were incubated with the appropriate secondary antibody (mouse or rabbit) 1:10,000 (*GE Healthcare *Life Sciences, UK), for 60 min over stirrer. Finally, membranes were washed again twice with TBST and once with TBS. To chemiluminescence analysis was used the ChemiDoc XRS+ system (Bio-Rad, California, USA) with Immobilon Forte Western HRP substrate (WBLUF0500, Millipore, Massachussets, USA). The Image Lab 6.0 software (Bio-Rad, California, USA) was used to analyse the acquired blot images and densitometric band quantification was performed by ImageJ 1.5k software (National Institute of Health, USA). Normalization of Western blots was performed using Ponceau S (Supplementary Fig. 1).

### Sandwich ELISA for GFAP levels

GFAP levels of plasma samples of patients and healthy controls were measured by sandwich enzyme-linked immunosorbent assay (ELISA) using a commercial kit (Human GFAP DuoSet ELISA Kit, DY2594-05, Lot P22599, R&D Systems, Minneapolis, USA) according to the manufacturer’s instructions. Briefly, plates, standards and reagents were prepared at room temperature (RT) following the protocol directions. Then, 100 µl of standards and samples were loaded into the 96-well plates and incubated 2 h at RT. With intervening washes, plates were successively incubated with 100 µl of detection antibody during 2 h, 20 min with 100 µl of streptavidin-HRP dilution, and 20 min with 100 µl of substrate solution. Finally, 50 µl of stop solution was added and gently mixed. Tests were performed in duplicated and an automated microplate reader (Biochrom ASYS UVM 340, Cambridge, UK) measured the optical density at 450 nm using Mikrowin 2000 software (Berthold Technologies, Germany). The GFAP concentration was normalised against total protein content measured by BCA protein assay (Pierce Chemicals, Rockford, IL).

### Statistics

Statistical analysis was performed using the GraphPad Prism 7 (GraphPad Software Inc., San Diego, CA, EUA). Mean age of both groups (schizophrenia and controls) was compared with the Mann–Whitney U test, and the differences between sex ratios were analysed with Fisher’s exact test. Each band of each blot was analysed individually and normalized based on the average of controls of each gel, whose results were converted in percentage and expressed in relative units. The unpaired two-tailed T-test was performed to compare the mean difference between schizophrenia group and control group. To determine whether there were significant differences among schizophrenia and control groups, one-way ANOVA followed by Bonferroni post-hoc test was done. The GFAP concentration measured by ELISA was calculated used a nonlinear regression method (R^2^ = 0.9989). Statistically significant results are achieved considering a *P* value ≤ 0.05.

## Results

A case–control study was performed to compare a group of patients with SZ, subdivided into two subgroups: FEP and CSZ, and a healthy control group. Both groups (SZ and control) were matched by age and gender, and there were no significant differences between groups regarding age (*P* = 0.3030), and gender (*P* = 1.0000) (Table 1).

One-way ANOVA test after *P* value adjustment was performed to compare illness onset and duration of illness between subgroups of treated patients. There were no significant differences between subgroups of patients treated with risperidone, aripiprazole, olanzapine, or clozapine (*P* > 0.05) (Supplementary Table 3). Clozapine dose equivalencies were also analysed between patients treated with antipsychotics, and no statistical significance was found (*P* > 0.05) (Supplementary Table 3). Concerning PANSS scores, the positive and negative subscales did not present statistically significant results among groups of patients. The PANSS general subscale showed significant results by one-way ANOVA (*P* = 0.0003), and after Bonferroni post-hoc test between groups: statistical differences between groups of FEP and CSZ on risperidone (41.22 ± 3.29 vs. 29.13 ± 2.52; *P* = 0.0068), groups of FEP and CSZ on olanzapine (41.22 ± 3.29 vs. 30.25 ± 1.86; *P* = 0.0179) and groups of FEP and CSZ on aripiprazole (41.22 ± 3.29 vs. 29.25 ± 1.83; *P* = 0.0075) were found. Scores of PANSS general also showed significant differences between groups of CSZ on clozapine and risperidone (39.44 ± 1.26 vs. 29.13 ± 2.52; *P* = 0.0113), groups of CSZ on clozapine and olanzapine (39.44 ± 1.26 vs. 30.25 ± 1.86; *P* = 0.0285) and groups of CSZ on clozapine and aripiprazole (39.44 ± 1.26 vs. 29.25 ± 1.83; *P* = 0.0126).

**Figure 2:**
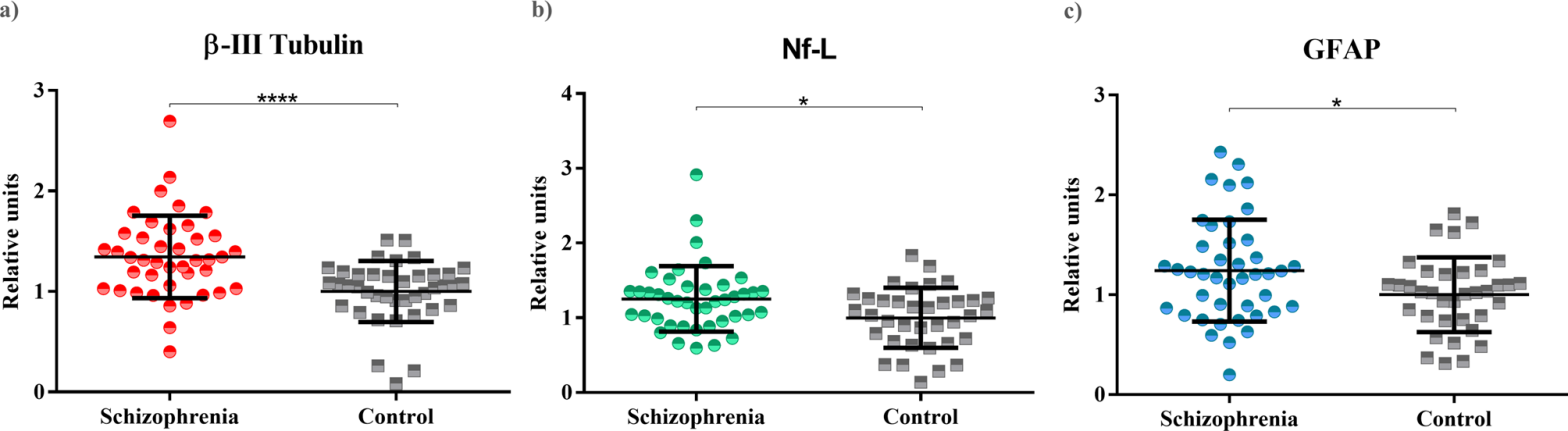
Plasma levels of β-III tubulin, Nf-L and GFAP proteins in patients with SZ (N = 42) and controls (N = 40). Scatterplots of plasma levels of structural proteins. (**a**) Levels of β-III tubulin of patients with schizophrenia and controls (*P* < 0.0001). (**b**) Levels of Nf-L of patients with schizophrenia and controls (*P* = 0.0101). (**c**) Levels of GFAP of patients with schizophrenia and controls (*P* = 0.0212).

In order to measure the potential neuronal damage in SZ patients vs healthy controls, the levels of β-III tubulin, Nf-L and GFAP were studied (Fig. 2) (Table 2). Levels of β-III tubulin were significantly higher in patients’ group compared with healthy controls (*P* < 0.0001). Levels of Nf-L and GFAP (measured both by ELISA and immunoblot) were also elevated in patients with SZ compared to healthy controls (*P* < 0.05).

**Table 2:** Summary one-way ANOVA and Bonferroni’s multiple comparisons tests results.

Parameter	FEP	Risperidone	Olanzapine	Aripiprazole	Clozapine	Controls
Plasma levels (relative units)						
***β-III tubulin***	1.12 ± 0.24	1.34 ± 0.22	1.34 ± 0.37	1.34 ± 0.67	1.58 ± 0.35	1.00 ± 0.30
P value Bonferroni post-hoc test	> 0.9999	0.0751	0.0837	0.0766	0.0001*	
One-way ANOVA	**F**_**(5,76)**_ = 5.437 P = 0.0002*					
**Nf-L**	1.08 ± 0.72	1.22 ± 0.19	1.21 ± 0.27	1.22 ± 0.30	1.52 ± 0.41	1.00 ± 0.40
P value Bonferroni post-hoc test	> 0.9999	0.9370	0.9370	0.9371	0.0071*	
One-way ANOVA	**F**_**(5,76)**_ = 2.469 P = 0.0403*					
**GFAP**	1.07 ± 0.59	1.20 ± 0.64	1.20 ± 0.21	1.21 ± 0.41	1.51 ± 0.57	1.00 ± 0.37
P value Bonferroni post-hoc test	> 0.9999	> 0.9999	> 0.9999	> 0.9999	0.0151*	
One-way ANOVA	F_(5,76)_ = 2.077 *P* = 0.0781					
**Ratio**						
**Neutrophils/lymphocytes**	2.59 ± 2.06	2.27 ± 0,96	2.59 ± 1.05	1.81 ± 0.66	4.96 ± 6.59	3.26 ± 3.45
P value Bonferroni post-hoc test	> 0.9999	> 0.9999	> 0.9999	> 0.9999	0.9162	
One-way ANOVA	F_(5,76)_ = 0.9717 *P* = 0.4406					

One-way ANOVA followed by Bonferroni post-hoc test: comparison of patient’s groups with control group.

*FEP* first-episode psychosis, *Nf-L* neurofilament light chain, *GFAP* glial fibrillary acidic protein.

*Statistical significance: *P* ≤ 0.05.

**Figure 3:**
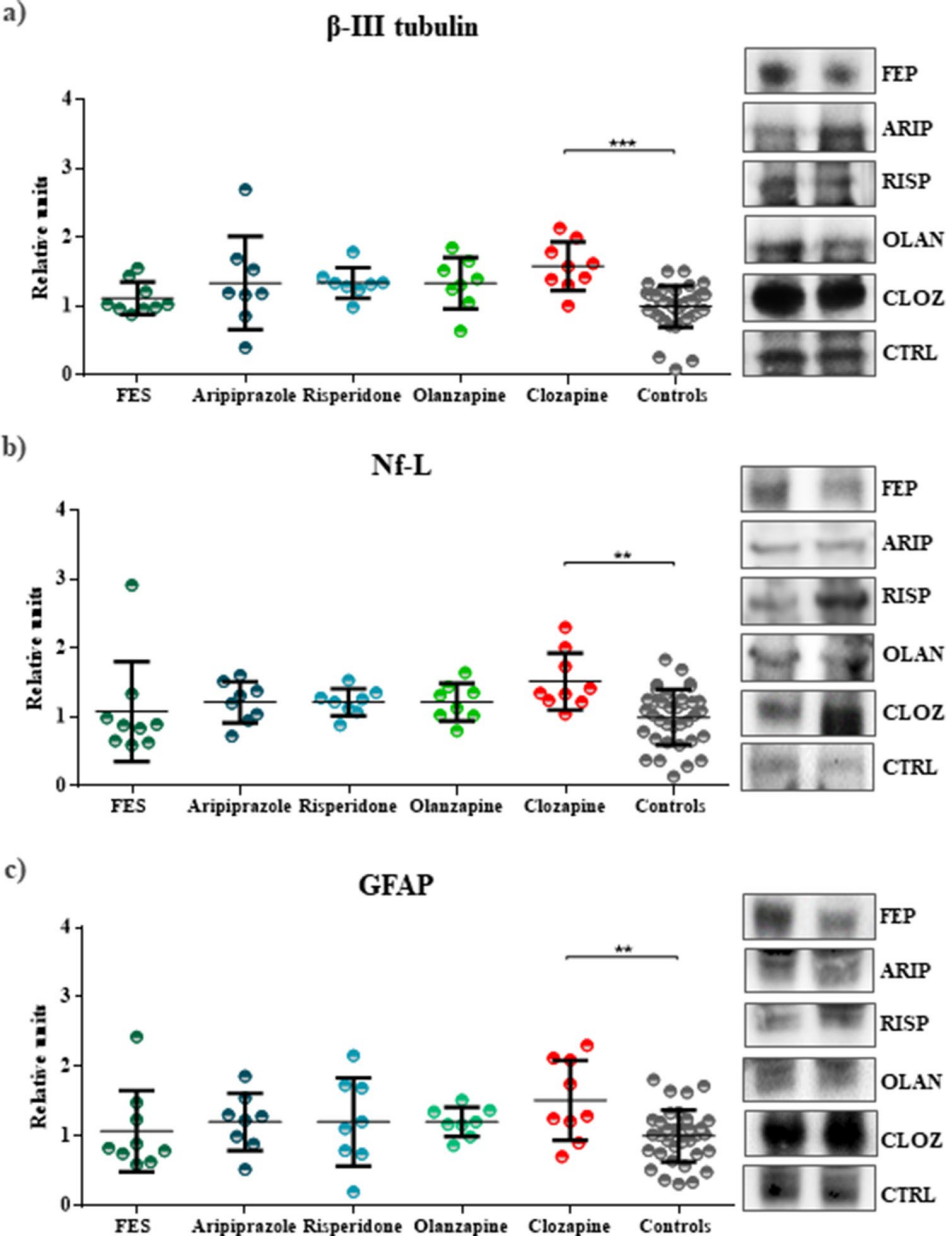
Plasma levels of β-III tubulin, Nf-L or GFAP in the subgroups of patients with SZ and controls. Scatterplots showing the levels of structural proteins by groups of patients with schizophrenia and the control group. (**a**) Scatterplot of levels of β-III tubulin that reveals statistical significance between clozapine-treated patients and control group (*P* = 0.0001). (**b**) Scatterplot of levels of Nf-L that showing a significant result between clozapine-treated patients and the control group (*P* = 0.0071). (**c**) Scatterplots of levels of GFAP, which demonstrates a significant correlation between clozapine-treated patients and controls (*P* = 0.0151). Full-length gels are presented in Supplementary Fig. 1.

Plasma levels of β-III tubulin between the five subgroups of patients with SZ and the healthy control group also showed significant differences (F_(5,76)=_5.437; *P* = 0.0002) (Table 2) (Fig. 3). Clozapine-treated patients presented statistical differences when compared with control group (1.58 ± 0.12 vs. 1.00 ± 0.05, *P* = 0.0001). Differences between the other groups of patients and controls were not statistically significant. Regarding Nf-L, similar results were found (F_(5,76)_ = 2.469; *P* = 0.0403), that is, patients treated with clozapine showed significant results (1.52 ± 0.14 vs. 0.99 ± 0.07, *P* = 0.0071), and no statistical significance was found between the other patients subgroups and the control group (Table 2) (Fig. 3). Finally, the levels of GFAP were once again slightly elevated in clozapine-treated patients (1.51 ± 0.19 vs. 1.00 ± 0.07, *P* = 0.0151), but no significant results were obtained for the other subgroups (Table 2) (Fig. 3). An ELISA assay was performed as an additional confirmation of GFAP levels. The results corroborated the immunoblot findings (F_(5,76)_ = 4.950; *P* = 0.0006), whose levels of GFAP were only increased on the clozapine-treated group (0.37 ± 0.13 vs. 0.23 ± 0.07, *P* < 0.0001) (Supplementary Table 4 and Supplementary Fig. 2).

Neutrophil–lymphocyte ratio (NLR) have been used as an indicator of inflammation. An elevation of NLR and neutrophils, and lower levels of lymphocytes were found in patients with SZ^44,45^. NLR is a strong predictor of systemic inflammation; NLR high values may be involved in the pathophysiology of SZ (inflammatory hypothesis), and also may be correlated with cognitive impairment^46,47^. To avoid the influence of immunity markers in our study, the NLR of patients and controls was calculated, and there were no statistically significant differences between groups (F_(5,76)=_0.9717; *P* = 0.4406).

## Discussion

The neurodegenerative course of SZ has been discussed since *Kraepelin* postulates*,* who described the unfavorable progression of SZ with a clear neurodegenerative trajectory^20,48^. However, two different heuristic approaches have been proposed to explain the pathophysiology of SZ, that could be described as the neurodevelopmental vs, neurodegenerative models^20^. Evidence suggests that the aetiology of SZ involves genetic and environmental factors that modify the neurodevelopment patterns during prenatal and perinatal stages, lasting at least until adolescence^8,24,49,50^. On the other hand, cognitive and functional progressive deterioration of patients with SZ also suggest a neurodegenerative process. Progressive morphological alterations (e.g. decreases in grey matter volume), number of relapses, DUP, and treatment with antipsychotic drugs seem to be involved in the degenerative processes seen in patients with SZ^4,26,51–54^. In this sense, the neurodegenerative model proposes a brain degeneration supported by clinical, biochemical (e.g. disruption of neurotransmitters systems) and neuroimaging findings (e.g. loss of cortical volume)^4,20,55,56^.

In this study, the levels of β-III tubulin, GFAP and Nf-L in plasma of patients with SZ, subdivided by treatment (aripiprazole, risperidone, olanzapine or clozapine) were analysed and compared with a group of healthy people. Only the subgroup of patients treated with clozapine showed significantly elevated plasma levels of β-III tubulin, GFAP and Nf-L. Essentially, our sample of SZ patients comprised three different types of patients: (a) a FEP group without treatment (analysed after their first-episode of psychosis); (b) a group of patients that after their first-episode either responded well to treatment and had full symptom remission, or patients who had a partial response to treatment with a slight clinical decline (risperidone, aripiprazole, olanzapine); and (c) a group of patients treated with clozapine that had had multiple psychotic episodes and did not respond previously to at least two different types of antipsychotics. Moreover, the illness progression and the clinical deterioration are more evident in the group treated with clozapine^57^. These different groups may give us an idea of ​​the three types of evolution of SZ^58,59^. Firstly, patients who respond appropriately to antipsychotics and are able to recover without significant cognitive damage. Secondly, patients who suffer new psychotic episodes, recovering badly from each of them and becoming a chronic process with a strong cognitive deterioration. Thirdly, patients who, for some reason, do not present neuronal damage after psychosis, despite having suffered several psychotic outbreaks^8,24^.

Most patients with SZ experience recurring psychotic relapses. The process of relapse, treatment failure, and incomplete recovery leads to a debilitating and chronic course of illness in many patients, and to persistent disturbances and deficits in perceptions, thought processes, and cognition. Although the majority of patients exhibit a severe pattern of deterioration, different degrees and temporal sequences may occur during SZ course.

Moreover, the results indicate that patients with SZ have neurodegenerative processes and we are able to detect them in peripheral samples (blood). However, the most interesting fact extracted from the analysis of β-III tubulin, Nf-L and GFAP proteins in CSZ patients treated with different antipsychotics is that it could be possible to predict the clinical course of the disease. Patients treated with clozapine presented high levels of β-III tubulin, Nf-L and GFAP compared with those who responded to first-line atypical antipsychotics. Patients treated with clozapine did not respond previously to at least two first-line antipsychotics, presenting a worse outcome. Two first-line treatment failures led to a longer DUP period, and therefore to a worse recovery, a greater number of hospitalizations and a greater cognitive impairment. This means that β-III tubulin, GFAP and Nf-L may be potential plasma biomarkers of neurodegeneration and progression in SZ and would help to classify and treat earlier CSZ patients with clozapine.

Abnormalities in cytoskeletal elements may be correlated with the pathophysiology of SZ^37^. Cytoskeleton structures of neuronal cells are composed by microtubules (MTs) such as β-tubulin, which play a role in neuronal morphology and polarity, signal events modulation, differentiation and viability of cells^37,60^. MTs are crucial to development and maintenance of neurons, and dysfunctions in their system contributes strongly to neurodegenerative processes^61^. The dynamic structure of the cytoskeleton of axons and dendrites is composed mainly by β-tubulin, which has main isotypes and each one present different functions^62^. The β-III tubulin is an isoform exclusively found in the nervous system^38^. Due to its crucial role in several functions in the nervous system, the disruption of MTs homeostasis has been associated with SZ, whose expression of tubulin isoforms is altered, indicating that it may become a potential biomarker in SZ^63^. Neurofilaments are also a major structural component of neurons and synapsis^40,64^. In neuronal degeneration (disruption of axonal membrane and cell death) the Nfs are released in a significant quantity into the interstitial fluid^65,66^. Specifically, the Nf-L plays a role in the shape maintenance of nerve cells, interacting with NR1 domain anchoring N-methyl-D-aspartate (NMDA) receptors in neurons’ plasma membranes^64^. Nf-L proteins are located in postsynaptic terminals, they are essential to neurotransmission and synaptic plasticity, suggesting they could be considered as potential biomarkers of synaptic degeneration^67^. High levels of Nf-L has been described in patients with familial Alzheimer's disease, being suggested as a marker of neurodegeneration^68^. Recently, it was reported that changes in serum Nf-L levels in patients with Alzheimer's disease were able to predict the onset of the disease years before the appearance of the first clinical symptoms^69^. In turn, the GFAP is an intermediate filament protein present in the cytoskeleton of astrocytes that provides functional and structural maintenance to neurons^70^. Evidence suggests that GFAP protein is involved in astrocytes functions that are crucial to neurogenesis, synaptic plasticity and reactive (astro)gliosis^71,72^. In the CNS, astrocytes are responsible for the homeostasis, and the loss of astrocytes is followed by neuronal damage^73^. Thus, the GFAP may be a biofluid marker of astroglial pathology and brain injury, correlating with neurodegeneration^70,74^. In the last few years, it has been described that these proteins can be detected in peripheral samples associated with neurodegeneration in different diseases^38–41^. For example, high levels of Nf-L were described in Alzheimer's disease^68,69^, and high levels of Nf-L and GFAP were associated with neuronal damage and progression of multiple sclerosis^75–78^.

Some limitations of the study will be mentioned here. Critical factors such as the self-report medication adherence, the impact of smoking or the hypothyroidism condition (in two patients treated with risperidone) on proteins levels were not contemplated. The common use of polytherapy in patients with schizophrenia in our site reduces the number of patients available per subgroups, which limits the statistical power due to the small sample size. In our opinion, a prospective study should be conducted to verify the effects of clozapine on the levels of proteins. Drugs such as ACE inhibitors, analgesics, statins, benzodiazepines or levothyroxine were administered concomitantly with antipsychotics to SZ patients in our sample. However, no correlation was found between these drugs and the levels of the three proteins in literature. On the other hand, potential confounding factors among patients such as age, duration of illness (chronicity), dose equivalents of atypical antipsychotics (mg of chlorpromazine) and inflammatory statement (NLR) were avoided in order to compare more efficiently the neurodegenerative processes associated with the chronic course of SZ.

## Conclusion

In conclusion, the neurodegenerative course of SZ has been a constantly debated question. However, the evidence remains uncertain, and the neurodegenerative model of SZ is poorly understood. Being aware of this gap, our objective was to identify potential plasma biomarkers that could be associated with molecular processes of neurodegeneration and course of SZ. Our results showed high levels of β-III tubulin, Nf-L and GFAP proteins in patients with CSZ compared to healthy subjects. This study demonstrates that a potential neurodegenerative process underlies the chronic course of SZ, specifically, in the clozapine-treated subgroup, who presented a worse prognosis, probably due to their refractory SZ condition. In conclusion, β-III tubulin, Nf-L and GFAP are potential biomarkers of neurodegeneration and progression in CSZ patients.

### Ethics approval and consent to participate

This study has been conducted in accordance with national and European legislation on clinical research, following international ethical recommendations, the Declaration of Helsinki and the Council of Europe regarding the Convention on Human Rights and Biomedicine. The study complied at all times with the requirements established in the Spanish legislation in the field of biomedical research, personal data protection and bioethics. This study was approved by the local ethics committee Galician Network of Research Ethics Committees, Registration code: 2019/120). Patients' ability to understand the voluntariness of the study was assessed by researchers employing clinical interview. The informed consent was obtained from all participants (all of them upper 18 years-old) The treating psychiatrists' opinion, based on the patients' clinical history, was also considered.

## Supplementary information

Supplementary Information.
