# Supplementary material for: Plasma β-III tubulin, neurofilament light chain and glial fibrillary acidic protein are associated with neurodegeneration and progression in schizophrenia
Source: Sci Rep. 2020 Aug 31;10:14271. doi: 10.1038/s41598-020-71060-4 (PMC7459108; doi:10.1038/s41598-020-71060-4)
Supplement: Supplementary file 1 — Supplementary Information. [file 41598_2020_71060_MOESM1_ESM.pdf]

Plasma β-III Tubulin, Neurofilament Light Chain and Glial Fibrillary Acidic Protein are Associated with Neurodegeneration and Progression in Schizophrenia

Daniela Rodrigues-Amorim[1¥], Tania Rivera-Baltanás[1¥,] María del Carmen Vallejo-Curto[1], Cynthia Rodriguez-Jamardo[1], Elena de las Heras[1], Carolina Barreiro-Villar[1], María Blanco-Formoso[1], Patricia Fernández-Palleiro[1], María Álvarez-Ariza[1], Marta López[1], Alejandro García-Caballero[2], José Manuel Olivares[1]*, Carlos Spuch[1]*

Supplementary table 1: Previous treatments of the clozapine-treated group.

Patient code	Current treatment	First treatment	Second treatment	Illness onset	Duration of illness
**E070**	**Clozapine**	Risperidone	Risperidone + Olanzapine	31	14
**E079**	**Clozapine**	Aripiprazole	Aripiprazole + Quetiapine	53	6
**E102**	**Clozapine**	Amisulpride	Olanzapine	20	9
**E117**	**Clozapine**	Risperidone	Risperidone + Haloperidol	25	27
**E128**	**Clozapine**	Risperidone	Risperidone + Amisulpride	30	30
**E175**	**Clozapine**	Zuclopentixol	Aripiprazole	22	32
**E176**	**Clozapine**	Haloperidol	Risperidone	30	11
**E178**	**Clozapine**	Aripiprazole	Aripiprazole + Clotiapine	21	3
**E179**	**Clozapine**	Risperidone	Risperidone + Quetiapine	23	23

Supplementary table 2: Equivalent amount of proteins (volume - µl) calculation by the a bicinchoninic acid assay (BCA).

Patient	SZ/Ctrl	Absorbance	Concentration µg/ml	Volume µl (10 µg)
**E113**	**FEP**	2,136	**4158,9**	**2**
**E118**	**FEP**	2,188	**4260,8**	**2**
**E123**	**FEP**	2,527	**4924,8**	**2**
**E130**	**FEP**	1,912	**3720,1**	**3**
**E135**	**FEP**	2,889	**6565,4**	**2**
**E136**	**FEP**	2,170	**4855,8**	**2**
**E140**	**FEP**	2,672	**6049,5**	**2**
**E167**	**FEP**	2,510	**5664,3**	**2**
**E170**	**FEP**	2,560	**5783,1**	**2**
**E074**	**Aripiprazole**	2,931	**5716,2**	**2**
**E075**	**Aripiprazole**	2,419	**2645,8**	**4**
**E093**	**Aripiprazole**	2,282	**2495,7**	**4**
**E119**	**Aripiprazole**	2,400	**2625,0**	**4**
**E122**	**Aripiprazole**	2,289	**2503,4**	**4**
**E126**	**Aripiprazole**	2,236	**2445,3**	**4**
**E131**	**Aripiprazole**	2,201	**2406,9**	**4**
**E134**	**Aripiprazole**	2,056	**2248,0**	**4**
**E088**	**Risperidone**	2,212	**2419,0**	**4**
**E114**	**Risperidone**	2,173	**2376,2**	**4**
**E115**	**Risperidone**	2,085	**4059,0**	**2**
**E132**	**Risperidone**	1,523	**1664,0**	**6**
**E141**	**Risperidone**	2,262	**2473,8**	**4**
**E143**	**Risperidone**	2,108	**2305,0**	**4**
**E150**	**Risperidone**	2,349	**2569,1**	**4**
**E163**	**Risperidone**	2,195	**2400,4**	**4**
**E086**	**Olanzapine**	2,218	**4319,5**	**2**
**E094**	**Olanzapine**	2,206	**2412,4**	**4**
**E112**	**Olanzapine**	2,236	**2445,3**	**4**
**E116**	**Olanzapine**	2,224	**2432,1**	**4**
**E121**	**Olanzapine**	2,218	**2425,6**	**4**
**E149**	**Olanzapine**	2,206	**2412,4**	**4**
**E160**	**Olanzapine**	2,212	**2419,0**	**4**
**E190**	**Olanzapine**	2,243	**2453,0**	**4**
**E070**	**Clozapine**	2,138	**4162,8**	**2**
**E079**	**Clozapine**	2,272	**4425,3**	**2**
**E102**	**Clozapine**	2,025	**3941,5**	**3**
**E117**	**Clozapine**	2,648	**5161,8**	**2**
**E128**	**Clozapine**	2,101	**4090,4**	**2**
**E175**	**Clozapine**	2,401	**5405,1**	**2**
**E176**	**Clozapine**	2,310	**5188,7**	**2**
**E178**	**Clozapine**	2,196	**4276,4**	**2**
**E179**	**Clozapine**	1,989	**3871,0**	**3**
**654**	**Control**	2,389	**4231,4**	**2**
**655**	**Control**	2,466	**4231,4**	**2**
**656**	**Control**	2,426	**4231,4**	**2**
**657**	**Control**	2,165	**4231,4**	**2**
**658**	**Control**	2,757	**4231,4**	**2**

**659**	**Control**	2,363	**4231,4**	**2**
**660**	**Control**	2,228	**4231,4**	**2**
**661**	**Control**	2,574	**4231,4**	**2**
**662**	**Control**	2,316	**4231,4**	**2**
**663**	**Control**	2,389	**4231,4**	**2**
**664**	**Control**	2,125	**4231,4**	**2**
**665**	**Control**	1,875	**4231,4**	**2**
**666**	**Control**	2,228	**4231,4**	**2**
**667**	**Control**	2,015	**4231,4**	**2**
**668**	**Control**	2,050	**4231,4**	**2**
**669**	**Control**	2,260	**4231,4**	**2**
**670**	**Control**	2,101	**4231,4**	**2**
**671**	**Control**	1,780	**4231,4**	**2**
**672**	**Control**	2,331	**4231,4**	**2**
**673**	**Control**	2,247	**4231,4**	**2**
**674**	**Control**	2,266	**4231,4**	**2**
**675**	**Control**	2,436	**4231,4**	**2**
**676**	**Control**	2,426	**4231,4**	**2**
**677**	**Control**	2,115	**4231,4**	**2**
**678**	**Control**	2,389	**4231,4**	**2**
**679**	**Control**	2,115	**4231,4**	**2**
**680**	**Control**	2,216	**4231,4**	**2**
**681**	**Control**	2,301	**4231,4**	**2**
**682**	**Control**	2,280	**4231,4**	**2**
**683**	**Control**	2,062	**4231,4**	**2**
**684**	**Control**	1,969	**4231,4**	**2**
**S254**	**Control**	2,720	**2975,7**	**3**
**S255**	**Control**	2,739	**2996,5**	**3**
**S256**	**Control**	2,634	**2881,4**	**3**
**S259**	**Control**	2,701	**2954,8**	**3**
**S261**	**Control**	2,683	**2935,1**	**3**
**S262**	**Control**	2,666	**2916,5**	**3**
**S264**	**Control**	2,634	**2881,4**	**3**
**S268**	**Control**	2,619	**2865,0**	**3**
**S288**	**Control**	2,780	**3041,4**	**3**

Legend: SZ – schizophrenia; Ctrl - Control; FEP - first-episode psychosis.

Supplementary table 3: Summary One-way ANOVA and Bonferroni's multiple comparisons tests results.

**Duration of illness**					
**ANOVA Summary**				**F= 0.1973**	**P=0.8974**
**Bonferroni's multiple comparison test**	**Mean Diff.**	**95% CI of diff.**	**Significance**	**Summary**	**Adjusted P Value**
Aripiprazole vs. Risperidone	-0.6250	-14.77 to 13.52	No	ns	>0.9999
Aripiprazole vs. Olanzapine	-1.500	-15.64 to 12.64	No	ns	>0.9999
Aripiprazole vs. Clozapine	-3.472	-17.22 to 10.27	No	ns	>0.9999
Risperidone vs. Olanzapine	-0.8750	-15.02 to 13.27	No	ns	>0.9999
Risperidone vs. Clozapine	-2.847	-16.59 to 10.90	No	ns	>0.9999
Olanzapine vs. Clozapine	-1.972	-15.72 to 11.77	No	ns	>0.9999
**Dose equivalencies**					
**ANOVA Summary**				**F= 2.413**	**P=0.0870**
**Bonferroni's multiple comparison test**	**Mean Diff.**	**95% CI of diff.**	**Significance**	**Summary**	**Adjusted P Value**
Aripiprazole vs. Risperidone	-101.7	-300.8 to 97.49	No	ns	0.9543
Aripiprazole vs. Olanzapine	85.83	-113.3 to 285.0	No	ns	>0.9999
Aripiprazole vs. Clozapine	-23.89	-217.4 to 169.7	No	ns	>0.9999
Risperidone vs. Olanzapine	187.5	-11.66 to 386.7	No	ns	0.0745
Risperidone vs. Clozapine	77.78	-115.8 to 217.3	No	ns	>0.9999
Olanzapine vs. Clozapine	-109.7	-303.3 to 83.83	No	ns	0.7157
**Illness onset**					
**ANOVA Summary**				**F=2.857**	**P=0.0556**
**Bonferroni's multiple comparison test**	**Mean Diff.**	**95% CI of diff.**	**Significance**	**Summary**	**Adjusted P Value**
Aripiprazole vs. Risperidone	-13.00	-26.97 to 0.9662	No	ns	0.0798
Aripiprazole vs. Olanzapine	-1.446	-14.97 to 12.08	No	ns	>0.9999
Aripiprazole vs. Clozapine	-4.905	-18.07 to 8.263	No	ns	>0.9999
Risperidone vs. Olanzapine	11.55	-1.969 to 25.08	No	ns	0.1315
Risperidone vs. Clozapine	8.095	-5.072 to 21.26	No	ns	0.5486
Olanzapine vs. Clozapine	-3.458	-16.15 to 9.236	No	ns	>0.9999

Legend: One-way ANOVA followed by Bonferroni post-hoc test: comparison between patients treated with antipsychotics *Statistical significance: $P \leq 0.05$.

Supplementary figure 1: Original western blots.

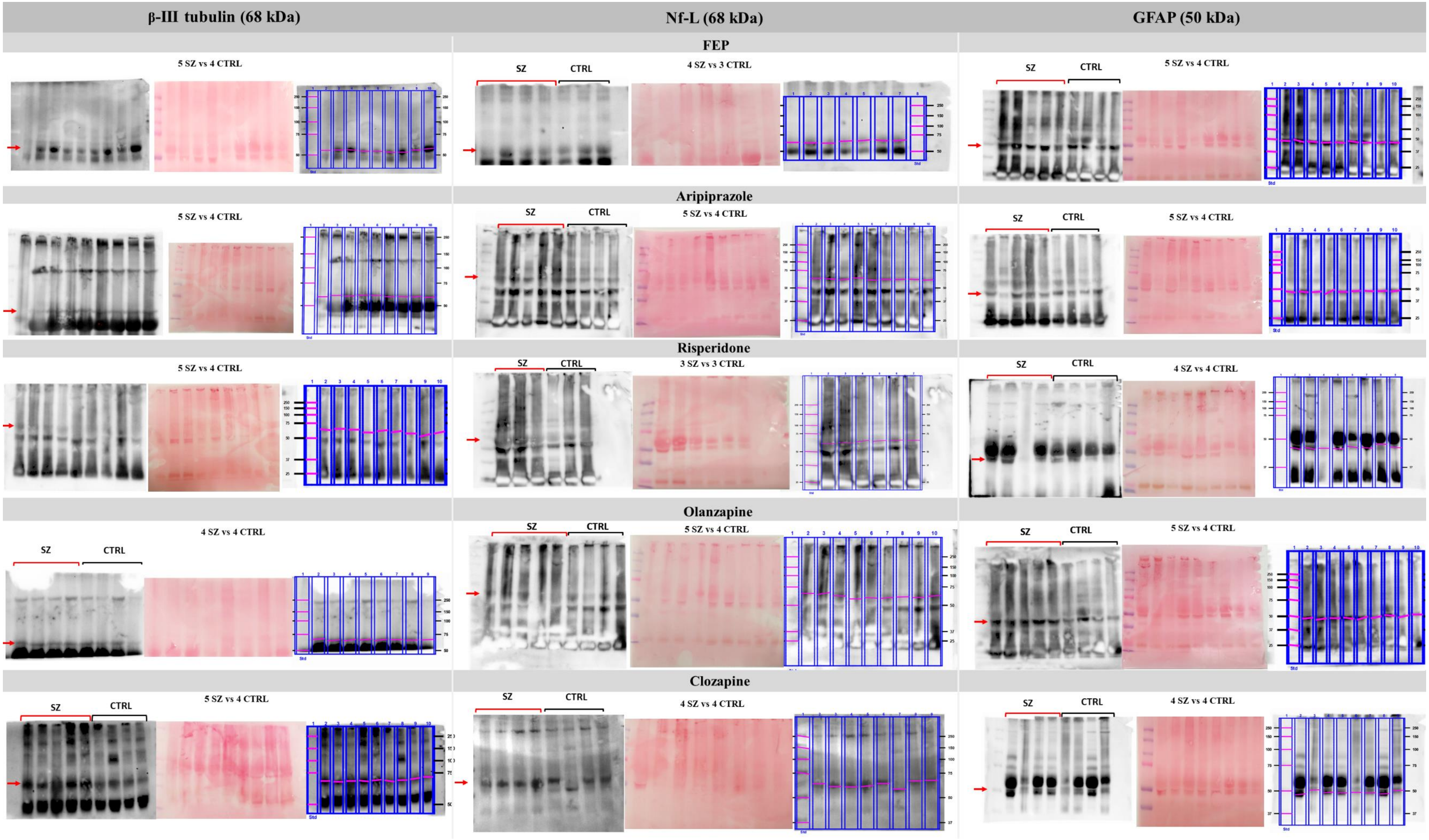

Supplementary table 4: Summary One-way ANOVA and Bonferroni's multiple comparisons tests results for GFAP ELISA.

GFAP					
**ANOVA Summary**				**F= 4.950**	**P=0.0006**
**Bonferroni's multiple comparison test**	**Mean Diff.**	**95% CI of diff.**	**Significance**	**Summary**	**Adjusted P Value**
FEP vs. Controls	-0,009998	-0,08676 to 0,06676	No	ns	> 0.9999
Aripiprazole vs. Controls	-0,02356	-0,1041 to 0,05702	No	ns	> 0.9999
Risperidone vs. Controls	-0,01657	-0,09715 to 0,06401	No	ns	> 0.9999
Olanzapine vs. Controls	0,002630	-0,07795 to 0,08321	No	ns	> 0.9999
Clozapine vs. Controls	-0,1412	-0,2180 to -0,06448	Yes	****	< 0.0001

Legend: One-way ANOVA followed by Bonferroni post-hoc test: comparison between patients treated with antipsychotics and controls *Statistical significance: $P \leq 0.05$.

Supplementary figure 2: Plasma levels of GFAP measured by ELISA.

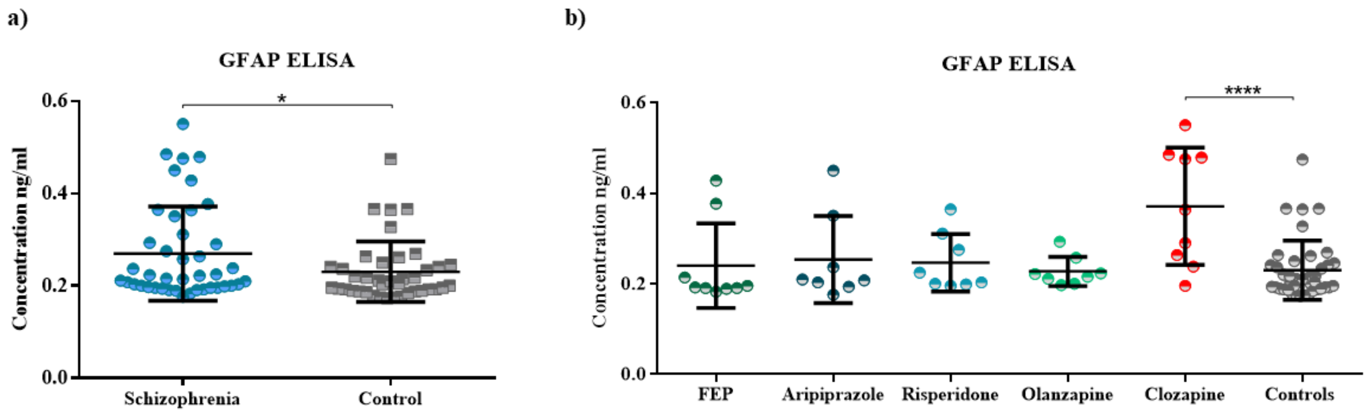

Legend: Scatterplots showing the plasma levels of GFAP. a) Scatterplot of levels of GFAP that reveals statistical significance between patients with schizophrenia and the control group using unpaired two-tailed T-test (P=0.0407). b) Scatterplot of levels of GFAP that showing a significant result between clozapine-treated patients and the control group using one-way ANOVA multiple comparison ($P<0.0001$).
